# An Empirical Modeling of Gate Voltage-Dependent Behaviors of Amorphous Oxide Semiconductor Thin-Film Transistors including Consideration of Contact Resistance and Disorder Effects at Room Temperature

**DOI:** 10.3390/membranes11120954

**Published:** 2021-12-01

**Authors:** Sungsik Lee

**Affiliations:** Department of Electronics Engineering, Pusan National University, Pusan 46241, Korea; sungsiklee@pusan.ac.kr; Tel.: +82-51-510-3123

**Keywords:** amorphous oxide semiconductor, thin-film transistor, degree of disorder, bias-dependent contact resistance, compact transistor model, empirical modeling, transfer characteristics

## Abstract

In this paper, we present an empirical modeling procedure to capture gate bias dependency of amorphous oxide semiconductor (AOS) thin-film transistors (TFTs) while considering contact resistance and disorder effects at room temperature. From the measured transfer characteristics of a pair of TFTs where the channel layer is an amorphous In-Ga-Zn-O (IGZO) AOS, the gate voltage-dependent contact resistance is retrieved with a respective expression derived from the current–voltage relation, which follows a power law as a function of a gate voltage. This additionally allows the accurate extraction of intrinsic channel conductance, in which a disorder effect in the IGZO channel layer is embedded. From the intrinsic channel conductance, the characteristic energy of the band tail states, which represents the degree of channel disorder, can be deduced using the proposed modeling. Finally, the obtained results are also useful for development of an accurate compact TFT model, for which a gate bias-dependent contact resistance and disorder effects are essential.

## 1. Introduction

Amorphous semiconducting materials such as amorphous Silicon and amorphous oxide semiconductors (AOSs) have been widely used as the channel layer for thin-film transistors (TFTs) [1,2,3]. In particular, it is believed that the AOS TFT has become one of the most promising candidates for futuristic electronics due to its high transparency and low temperature processability [3,4,5]. With a low temperature process, the AOS film is more likely to be in amorphous phase; thus, the inevitable presence of localized traps (e.g., deep and tail states) is associated with structural disorder in the amorphous phase [2,6]. This additionally alludes to a poor quality of metal contacts at the source and drain [1,2,7]. It is known that these non-ideal properties have a significant influence on the electrical performances of the AOS TFTs [2,3,8]; for example, the field-effect mobility of AOS TFTs is inversely proportional to the density of the localized tail states, while poor contact can lead to a higher contact resistance, and thus a lower mobility [9]. In addition, the contact resistance is typically extracted with a transmission line method (TLM), yielding a constant value without a gate-bias dependency [10]. Therefore, it is necessary to analyze and model the correlation between the transistor characteristics and those parasitic properties which include gate bias-dependent contact resistance.

In this paper, to capture gate bias-dependent contact resistance (*R_C_*) and Intrinsic channel conductance (*G_int_*) in the AOS TFT, an empirical method is proposed based on transfer characteristics measured for the two different AOS TFTs, as the parasitic effects are reflected in the current-voltage characteristics of the transistors. For this, we examine three fabricated In-Ga-Zn-O (IGZO) TFTs. From them a pair of TFTs, chosen from three examined TFTs (i.e., three pairs are available as three combinations made from three examined TFTs), is used to retrieve the gate voltage-dependent *R_C_* and *G_int_* while applying an analytical expression derived from the current–voltage relations of TFTs. Based on the retrieved trend of the *R_C_* vs. *V_GS_*, which is modelled with a power law, the *G_int_* can also be accurately extracted and modelled, yielding the characteristic energy of band tail states as a measure of disorders (assuming the dominance of the trap-limited conduction). From these results, it is found that the gate-bias dependencies of both *R_C_* and *G_int_* are well explained with a power-law function. Finally, it is believed that the presented results could be useful for an accurate compact TFT model, where the gate-bias dependencies of the contact resistance and disorder effects are crucial.

## 2. Materials and Methods

**Figure 1:**
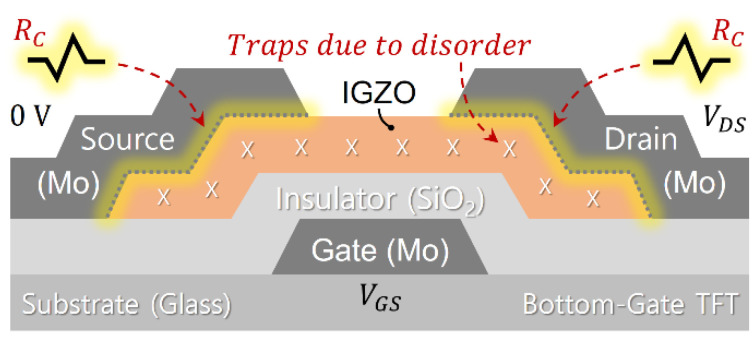
Schematic cross-sectional view of the examined IGZO TFT describing the contact resistance (*R_C_*) and disorder (traps) within the IGZO channel layer.

In this work, we examined AOS TFTs where amorphous IGZO is incorporated as the AOS material, with disorder in the amorphous phase (see Figure 1). Following a typical bottom-gate TFT process as reported in [11,12] for the deposition of the IGZO layer on a glass substrate, an IGZO ceramic target was employed for RF sputtering with Ar plasma. During this sputtering process, the oxygen partial pressure was kept at a low level (e.g., 5%), followed by backend processes such as thermal annealing, patterning, metallization with Mo for the electrodes (i.e., source, drain, and gate), and passivation. Using this process, we prepared three IGZO TFTs with three different channel lengths (*L*_1,_ *L*_2,_ *L*_3_). Here, the channel widths for those TFTs remain the same (*W = W*_1_ *= W*_2_ *= W*_3_).

Using the three fabricated TFTs (TFT-1, TFT-2, TFT-3), the transfer characteristics, i.e., the drain current (*I_DS_*) vs. gate voltage (*V_GS_*), were measured at room temperature (300 K), which formed the basis for the proposed empirical modeling. The channel geometrical details of the examined TFTs are summarized for the three possible combinations out of three TFTs in Table 1. Along with the examined TFTs, empirical modeling was performed with relevant mathematical formulations, as explained in the following sections.

## 3. Results

### 3.1. Mathematical Formulations for AOS TFTs

Considering the channel disorder and contact resistance, the transfer characteristics in the linear regime, i.e., *I_DS_* vs. *V_GS_*, of n-channel AOS TFTs can be represented as follows:

(1) $$I_{DS}=K_{n}\frac{W}{L}{\left(V_{GS}-V_{T}\right)}^{1+\alpha_{cm}}\left(V_{DS}-2R_{C}I_{DS}\right).$$

where *K_n_* is a pre-factor, *V_T_* is the threshold voltage, *α_cm_* is an exponent related to the conduction mechanisms (e.g., trap-limited conduction or percolation conduction), *V_DS_* is the drain voltage, and *R_C_* is the contact resistance. Note that in Equation (1), the physical meaning of *α_cm_* can be changed when the dominant conduction mechanism is determined from the trap-limited conduction (*α_cm_* ≡ *α_t_*, a trap-related exponent) or the percolation conduction (*α_cm_* ≡ *α_p_*, a percolation-related exponent) [13,14,15]. For example, depending on the position of the Fermi level (*E_F_*), the dominant conduction mechanism is determined [13,15]. In the present study, we presume that the *E_F_* is well below the *E_C_* for the given range of the *V_GS_* [15]. Thus, assuming that the trap-limited conduction is dominant, *α_cm_* in Equation (1) can now be related to the traps, thus *α_cm_* = *α_t_*. Based on this assumption, *K_n_* and *α_t_* are further defined, respectively, as follows:

(2) $$K_{n}=\mu_{n}\frac{C_{ox}^{1+\alpha_{t}}}{Q_{ref}^{\alpha_{t}}},$$

(3) $$\alpha_{t}=\frac{kT_{t}}{kT}\;or\;\frac{T_{t}}{T},$$

where *μ_n_* is the electron mobility, *C_ox_* is the gate insulator capacitance per area, *Q_ref_* is a reference charge-density per area, *kT_t_* is the characteristic energy of tail states, and *kT* is the thermal energy (Here, *k* is Boltzmann’s constant). Note that for a given temperature (*T*), Equation (3) can be rewritten as *kT_t_* = *α_t_ kT* or *T_t_* = *α_t_ T*. Here, a larger value of *kT_t_* or *T_t_* means a higher degree of disorder at a fixed *T* [15,16]. As can be seen in Equations (1)–(3), the formula for the drain current is strongly related to both the contact resistance and the disorder through *α_t_* as a function of *kT_t_* at a given temperature (*T*). In other words, Equation (1), where *α_t_* is included, is more general compared to the textbook, where an ideal case with *R_C_* = *α_t_* = 0 is only covered for a perfect crystalline semiconductor; hence, the universality of Equation (1).

**Figure 2:**
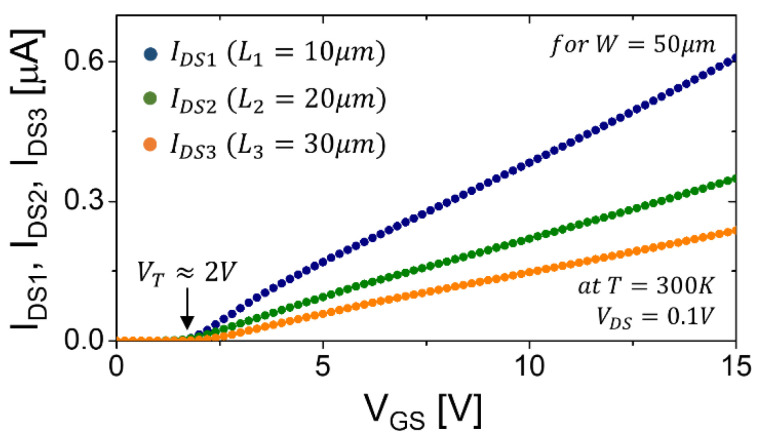
Measured transfer characteristics for *V_DS_* = 0.1 V of the fabricated IGZO TFT with three different *L* (see Table 1).

With Equation (1), the measured transfer characteristics in the linear regime (i.e., *V_GS_–V_T_ >> V_DS_* = 0.1 V) can be explained rather than using the ideally linear equation for *R_C_* = *α_t_* = 0. Indeed, as can be seen in Figure 2, the curvature in the above threshold region (at *V_GS_ > V_T_*) looks somewhat like a root function; thus *R_C_* ≠ 0 and *α_t_* ≠ 0. Note that V_T_ is extracted with the second derivative method rather than a linear extrapolation [16]. This suggests the presence of contact resistance and disorder (i.e., tail states); thus, the *R_C_* and *α_t_* should be extracted in order to capture that behavior, as seen in Figure 2. In the following two sub-sections, the retrieval procedure for R_C_ and α_t_ is shown in detail.

### 3.2. Contact Resistance and Transfer Characteristics of two TFTs

To extract the contact resistance (*R_C_*), we need to derive the respective formula based on Equation (1). For the first combination with TFT-1 and TFT-2, for example, referring to Table 1, the current-voltage relations are as follows:

(4) $$I_{DS1}=K_{n}\frac{W_{1}}{L_{1}}{\left(V_{GS}-V_{T}\right)}^{1+\alpha_{t}}\left(V_{DS}-2R_{C}I_{DS1}\right),$$

(5) $$I_{DS2}=K_{n}\frac{W_{2}}{L_{2}}{\left(V_{GS}-V_{T}\right)}^{1+\alpha_{t}}\left(V_{DS}-2R_{C}I_{DS2}\right).$$

Here, it is notable that *I_DS_* changes with varying *W/L*, while other parameters, including *R_C_*, are given or constant regardless of *W/L*. Note that the terms $\left(V_{DS}-2R_{C}I_{DS1}\right)$ and $\left(V_{DS}-2R_{C}I_{DS2}\right)$ in Equations (4) and (5) are called effective drain voltage, which can also be extracted using *R_C_*. From Equations (4) and (5), we can take their ratio as

(6) $$\frac{I_{DS1}}{I_{DS2}}=\frac{W_{1}L_{2}\left(V_{DS}-2R_{C}I_{DS1}\right)}{W_{2}L_{1}\left(V_{DS}-2R_{C}I_{DS2}\right)}.$$

Equation (6) can be expressed for the *R_C_* as follows:

(7) $$R_{C}=\frac{\left(W_{1}L_{2}I_{DS2}-W_{2}L_{1}I_{DS1}\right)}{2I_{DS1}I_{DS2}\left(W_{1}L_{2}-W_{2}L_{1}\right)}V_{DS}\;\left[\Omega \right].$$

Note that Equation (7) is an analytical expression of the *R_C_* for the first pair of TFTs (i.e., TFT-1 and TFT-2). If *W*_1_ = *W*_2_, Equation (7) is reduced as

(8) $$R_{C}=\frac{\left(L_{2}I_{DS2}-L_{1}I_{DS1}\right)}{2I_{DS1}I_{DS2}\left(L_{2}-L_{1}\right)}V_{DS}\;\left[\Omega \right].$$

Similarly, for the second combination, TFT-2 and TFT-3 for *W*_2_ *= W*_3_, Equation (8) is rewritten as

(9) $$R_{C}=\frac{\left(L_{3}I_{DS3}-L_{2}I_{DS2}\right)}{2I_{DS2}I_{DS3}\left(L_{3}-L_{2}\right)}V_{DS}\;\left[\Omega \right].$$

As the third pair, Equation (8) for TFT-3 and TFT-1 for *W*_3_ *= W*_1_ is reconstructed as

(10) $$R_{C}=\frac{\left(L_{3}I_{DS3}-L_{1}I_{DS1}\right)}{2I_{DS1}I_{DS3}\left(L_{3}-L_{1}\right)}V_{DS}\;\left[\Omega \right].$$

Note that Equations (8)–(10) can also be normalized with the given channel width (*W*), giving the normalized contact resistance (*r_c_*) as $r_{c}=R_{C}W\;\left[\Omega \cdot \mathrm{cm}\right].$

**Figure 3:**
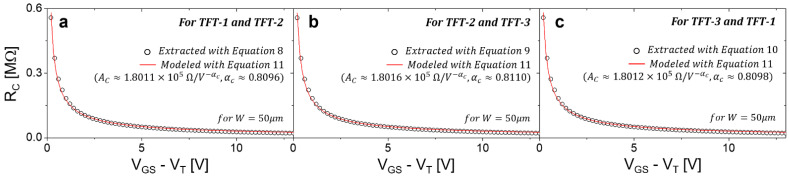
The retrieved *R_C_* as a function of *V_GS_-V_T_* for three possible combinations, which are the three cases: (**a**) TFT-1 and TFT-2; (**b**) TFT-2 and TFT-3; (**c**) TFT-3 and TFT-1. Here, the modeled results indicating the values of the model parameters (*A_C_* and *α_c_*) for each case are also shown.

Now, Equations (8)–(10) can be applied to obtain *R_C_* for those three combinations, respectively, along with the measured transfer characteristics seen in Figure 2. Figure 3 shows *R_C_* as a function of *V_GS_-V_T_*, along with the modeled plots. As can be seen, they show good agreement for all three difference cases. For these modelings, we employed a power-law function:

(11) $$R_{C}=A_{C}{\left(V_{GS}-V_{T}\right)}^{-\alpha_{c}}\;\left[\Omega \right],$$

where *A_C_* is a pre-factor and *α_c_* is an exponent. As indicated in Figure 3, the extracted values of *A_C_* and *α_c_* for all three cases are approximately 1.8 *×* 10^5^ $\Omega /V^{-\alpha_{c}}$ and 0.81, respectively.

### 3.3. Intrinsic Channel Conductance and Tail States

First, the ratio between the *I_DS_* and $\left(V_{DS}-2R_{C}I_{DS}\right)$ of Equation (1) is normalized with *W/L*. From this, an empirical expression of the intrinsic channel conductance (*G_int_*) is given while replacing the term *R_C_* with Equation (11), as follows:

(12) $$G_{int}=\frac{I_{DS}}{\left(V_{DS}-2A_{C}{\left(V_{GS}-V_{T}\right)}^{-\alpha_{c}}I_{DS}\right)\left(W/L\right)}.$$

The remaining term in the right-hand-side of Equation (1) is then given independently as

(13) $$G_{int}=K_{n}{\left(V_{GS}-V_{T}\right)}^{1+\alpha_{t}}.$$

**Figure 4:**
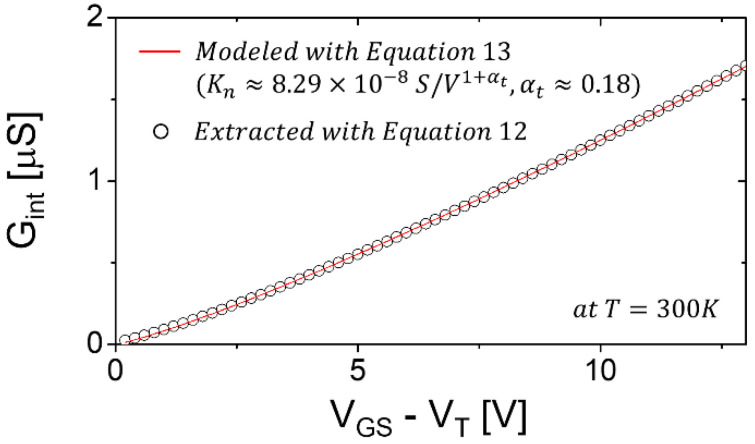
Plot of *G_int_* as a function of *V_GS_-V_T_*; the retrieved values of *K_n_* and *α_t_* are indicated. In particular, the *α_t_* is 0.18; thus, *kT_t_* ≈ 4.7meV (i.e., *T_t_* ≈ 54 K).

By applying Equation (12) using the results seen in Figure 2 and Figure 3, *G_int_* vs. *V_GS_-V_T_* can be extracted; meanwhile, Equation (13) is applied separately, yielding the values of *K_n_* and *α_t_*, respectively (see Figure 4). Note that in Figure 4 the *G_int_* is provided with the *R_C_* effect removed and the channel geometry (*W*/*L*) normalized, and is thus common to all examined TFTs.

## 4. Discussion

As mentioned in the Section 3.1, the measured transfer characteristics of the AOS TFTs can be explained with Equation (1) rather than the ideally-linear equation for *R_C_* = *α_t_* = 0. Indeed, as can be seen in Figure 2, it was found that the curvature in the above-threshold region (at *V_GS_ > V_T_*) looks slightly like a root function, implying the presence of contact resistance and disorder (i.e., *R_C_* ≠ 0 and *α_t_* ≠ 0). 

Regarding the effects of contact resistance, *R_C_* was extracted, as seen in Figure 3, by applying Equations (8)–(10) to the transfer characteristics of three pairs of TFTs (see Figure 2). As can be seen in Figure 3, it is obvious that the extracted results have been well-matched with the proposed model using a power-law. Here, it was found that it decays with increasing V_GS_. This is because the contact resistance is reduced by narrowing of the Schottky barrier due to a higher gate bias [2,17]. In keeping with this, the results modeled using Equation (11) showed good agreement in all three cases. The retrieved values of the *R_C_* model parameters are listed in the first row of Table 2. As can be seen in Figure 3 and Table 2, the values of the *A_C_* and *α_c_* for all three cases are approximately very similar to each other, with discrepancy negligible at less than 1%. This suggests that the *R_C_* extraction method proposed here is accurate and consistent. Note that the extracted *R_C_* trend for different pairs of TFTs should be the same in principle, and very similar practically as long as the same initial fabrication process was applied and the same ambient conditions were maintained.

Using the retrieved model parameters for *R_C_*, the intrinsic channel conductance (*G_int_*) was then accurately extracted using Equation (12). In other words, we were able to remove the effect of *R_C_* for accurate extraction of *G_int_*. As seen in Figure 4, it is clear that good agreement between the extracted and modeled results was achieved. Here, it was found that *G_int_* increased without any root-function behavior. However, it exhibited slightly parabolic behavior, with an exponent > 1 using the power law. From this result, it was found that the trend of *G_int_* could be modelled with a power law with the exponent 1.18 (i.e., Equation (13) with 1 *+ α_t_* for *α_t_* = 0.18). While the crystalline material-based transistor has the exponent of unity, in our case, the retrieved value of the exponent, i.e., 1.18, was found to be slightly greater than unity. This implies that the channel material is non-crystalline, and thus a disorder. As seen in Table 2, *kT_t_* is 4.7 meV < *kT* at 300 K, which is consistent with the literature [16,18]. This suggests that the degree of disorder is less than amorphous Silicon, with *kT_t_ > kT*. As for a physical interpretation, the conduction band of the AOS, e.g., IGZO, is composed of spherical orbitals (i.e., s orbitals) of metal cations; thus, the AOS is insensitive to the bonding angle disorder, whereas amorphous Silicon has strong bonding directivity due to the sp3 orbitals of its conduction band [18,19,20]. However, the AOS still has a bonding distance error in the amorphous phase, alluding to the existence of localized traps associated with band tail states, which can be estimated using extraction methods based on the current-voltage and capacitance-voltage characteristics [18,21]. In order to minimize these localized traps, process conditions can be optimized in terms of the AOS target compositions for the sputtering process, oxygen partial pressure, and annealing temperature [1,3,22].

Consequently, the presented empirical model for the transfer characteristics, in which the gate-bias dependencies of the contact resistance and disorder effects are considered, could be easily added into a compact TFT model thanks to its simplicity.

## 5. Conclusions

In this paper, we provided an empirical model to explain gate bias-dependent contact resistance and disorder effects in AOS TFTs at room temperature. As an intermediate outcome, we were also able to obtain the gate-bias dependency of the intrinsic channel conductance where the disorder effects were viewed after removal of the parasitic effect due to the contact resistance. As the first step, from the measured transfer characteristics of a pair of the IGZO TFTs, the contact resistance was extracted as a function of gate voltage. Here, its analytical expression, derived from the current–voltage relations of two TFTs (i.e., a pair of TFTs), was derived and applied for its retrieval. Interestingly, it was found to follow a simple power law, giving the values of the pre-factor and exponent. In addition, these values were found to be approximately the same for all three pairs examined here. This allowed accurate extraction of the gate bias-dependent intrinsic channel conductance as modelled using a power law. From this analysis, assuming the domination of trap-limited conduction, it was shown that the characteristic energy and temperature of the band tail states could be estimated as a measure of the degree of disorder, which is consistent with the literature. Consequently, the presented results could be useful for the development of an accurate compact TFT model, in which the gate bias-dependent behaviors of the contact resistance and disorder effects are crucial.

## Figures and Tables

**Table 1:** Summary of channel geometrical details of three examined IGZO TFTs.

Examined IGZO TFTs	Channel Length (*L*)	Channel Width (*W*)
TFT-1	*L*_1_*=* 10 μm	*W =* 50 μm (*common*) (*W = W*_1_ *= W*_2_ *= W*_3_)
TFT-2	*L*_2_*=* 20 μm	
TFT-3	*L*_3_*=* 30 μm	

**Table 2:** Summary of model equations and parameters for the same *W* at *T* = 300 K.

Non-Ideal Effects	Model Equations	Model Parameters
Contact Resistance	$R_{C}=\frac{\left(L_{2}I_{DS2}-L_{1}I_{DS1}\right)}{2I_{DS1}I_{DS2}\left(L_{2}-L_{1}\right)}V_{DS}$ (for example of TFT-1 and TFT-2) $R_{C}=A_{C}{\left(V_{GS}-V_{T}\right)}^{-\alpha_{c}}\;\left[\Omega \right]\;r_{c}=R_{C}W\;\left[\Omega \cdot \mathrm{cm}\right]$	*A_C_ ≈* 1.8 × 10^5^ $\Omega /V^{-\alpha_{c}}$ *for W =* 50 μm *α_c_ ≈* 0.81
Disorder (Traps)	$G_{int}\equiv \frac{I_{DS}}{\left(V_{DS}-2R_{C}I_{DS}\right)W/L}\;G_{int}=K_{n}{\left(V_{GS}-V_{T}\right)}^{1+\alpha_{t}}.$	*K_n_ ≈* 8.29 × 10^−8^ $S/V^{1+\alpha_{t}}$ *α_t_* ≈ 0.18 *kT_t_ ≈* 4.7 meV i.e., *T_t_ ≈* 54 K
